# Draft Genome Sequence of the Bacterium *Comamonas aquatica* CJG

**DOI:** 10.1128/genomeA.01186-16

**Published:** 2016-11-03

**Authors:** Wenkui Dai, Yutong Zhu, Xiqian Wang, Nazgul Sakenova, Zhenyu Yang, Hongjuan Wang, Guoqiao Li, Jiajing He, Deyu Huang, Yongfei Cai, Weizheng Guo, Qian Wang, Tingting Feng, Quanshui Fan, Tianling Zheng, Aidong Han

**Affiliations:** a State Key Laboratory for Cellular Stress Biology, School of Life Sciences, Xiamen University, Xiamen, China; b BGI, Shenzhen, China; c Center for Qinhao Research and The First Hospital Affiliated, Guangzhou University of Chinese Medicine, Guangzhou, China; d Chendu Military Region’s Center for Disease Control and Prevention, Chendu, China

## Abstract

A Gram-negative bacterial strain, *Comamonas aquatica* CJG, absorbs low-density lipoprotein but not high-density lipoprotein in serum. Here, we report its draft genomic sequence of 3,764,434 bp, containing total 3,425 genes, 27% of which encode proteins for metabolism and energy conversion, and it is 30% identical to the genome of *Comamonas testosteroni*.

## GENOME ANNOUNCEMENT

Through our effort to examine possible pathogens in water sources, a Gram-negative bacterial strain, *Comamonas aquatica* CJG, was isolated from Daguan River in Kuming, China, in 1963 (1, 2). *C. aquatica* is like a slightly curved rod, with two to eight flagella. Its liquid culture grows at 37°C with light-red color and a foul smell. It is resistant to several antibiotics, including penicillin, cephalosporin, and bacitracin, but it is highly sensitive to chloramphenicol and tetracycline (1). Interestingly, *C. aquatica* can absorb serum low-density lipoprotein (LDL) and very low-density lipoprotein (VLDL) but not high-density lipoprotein (HDL) (1, 2).

This strain was originally named *Pseudomonas* sp. 16Zu, since it was very close to the genus *Pseudomonas* biochemically and morphologically. Based on 16S rRNA gene sequence, we found that this strain is in fact quite different from *Pseudomonas* but rather is 99% identical to a lesser-known species, *Comamonas aquatica* (3, 4). It is also 96% identical to *Comamonas testosteroni* (5, 6). We therefore renamed this strain *Comamonas aquatica* CJG.

We then sequenced its whole genome by Illumina HiSeq 2000 using its DNA sample prepared by a commercial bacterial genomic extraction kit. To build libraries, the genomic DNA was sonicated with Covaris or Bioruptor to produce fragments of <500 bp in size, filled up and extended by Klenow DNA polymerase, and ligated to T-vector. Sequencing of this library with inserts of 150 to 500 bp produced 781 Mb paired-end reads (~200-fold coverage), which were filtered by the NGS QC toolkit (7) to generate clean data of 700 Mb. The clean data combined with another data set generated similarly using a library of 2,000 bp (350 Mb) were assembled in SOAP*denovo* (8). The final assembly has 136 scaffolds, with an *N*_50_ length of 284,677 bp and maximum length of 669,486 bp. The genome was annotated using the databases of COG, Swiss-Prot, KEGG, and TrEMBL (9–12).

The draft genome of the *C. aquatica* CJG strain is 3,764,434 bp. The G+C content is 64.4%. A total 3,425 genes of about 87% of the genome are annotated, encoding 3,125 proteins. Those responsible for metabolism and energy conversion account for 27% of the total genes, and about 470 genes are related to replication, transcription, translation, and trafficking. The bacterial strain also harbors 111 genes, functioning in protein posttranslational folding, modification, and homeostasis. About 145 genes encode signal transduction components and virulence factors, including EnvZ, KdpE, ComE, and CheA. Interestingly, the strain contains 209 functional unknown proteins. Whether these contribute to LDL absorbance remains to be determined.

A comparison of the *C. aquatica* CJG genome with the nucleotide database of all other bacterial genomes in GenBank using BLAST (13) indicated that the genome shows the highest similarity (*E* value ≤E^-5^) to *Comamonas testosteroni* (99 out of 320 contigs). The second best match is *Delftia acidovorans* (47 out of 320 contigs). Several other *Acidovorax* and *Alicycliphilus* strains also have some similarity (12 to 14 out of 320 contigs). All these genera belong to the *Comamonadaceae* family.

### Accession number(s).

This genome has been deposited in GenBank/ENA/DDBJ under accession number CP016603.

## References

[1] Zhu Y, Sheng J, Li C, Lin W, Li G, Wang L, Xu S, Yang R, You P, Gao C 1986 A *Pseudomonas*-like bacterial strain that induces agglutination of human and various animal sera. J Kunmei Med Collège 7:10–17. (In Chinese.)

[2] Gao C, Yuan X, Guo F, Zhu Y 1989 Application of *Pseudomonas*-like bacterial strain on the absorption of hyperlipidemic blood. Chin J Lab Med 12:157–158. (In Chinese.)

[3] Wauters G, De Baere T, Willems A, Falsen E, Vaneechoutte M 2003 Description of *Comamonas aquatica* comb. nov. and *Comamonas kerstersii* sp. nov. for two subgroups of *Comamonas terrigena* and emended description of *Comamonas terrigena*. Int J Syst Evol Microbiol 53:859–862. doi:10.1099/ijs.0.02450-0. 12807213

[4] Moissl C, Osman S, La Duc MT, Dekas A, Brodie E, Desantis T, Venkateswaran K 2007 Molecular bacterial community analysis of clean rooms where spacecraft are assembled. FEMS Microbiol Ecol 61:509–521. doi:10.1111/j.1574-6941.2007.00360.x. 17655710

[5] Bathe S, Hausner M 2006 Design and evaluation of 16S rRNA sequence based oligonucleotide probes for the detection and quantification of *Comamonas testosteroni* in mixed microbial communities. BMC Microbiol 6:54. doi:10.1186/1471-2180-6-54. 16772028 PMC1526739

[6] Ma YF, Zhang Y, Zhang JY, Chen DW, Zhu Y, Zheng H, Wang SY, Jiang CY, Zhao GP, Liu SJ 2009 The complete genome of *Comamonas testosteroni* reveals its genetic adaptations to changing environments. Appl Environ Microbiol 75:6812–6819. doi:10.1128/AEM.00933-09. 19734336 PMC2772449

[7] Patel RK, Jain M 2012 NGS QC toolkit: a toolkit for quality control of next generation sequencing data. PLoS One 7:e30619. doi:10.1371/journal.pone.0030619. 22312429 PMC3270013

[8] Luo R, Liu B, Xie Y, Li Z, Huang W, Yuan J, He G, Chen Y, Pan Q, Liu Y, Tang J, Wu G, Zhang H, Shi Y, Liu Y, Yu C, Wang B, Lu Y, Han C, Cheung DW, Yiu SM, Peng S, Xiaoqian Z, Liu G, Liao X, Li Y, Yang H, Wang J, Lam TW, Wang J 2012 SOAP*denovo*2: an empirically improved memory-efficient short-read *de novo* assembler. GigaScience 1:18. doi:10.1186/2047-217X-1-18. 23587118 PMC3626529

[9] Bairoch A, Apweiler R 2000 The Swiss-Prot protein sequence database and its supplement TrEMBL in 2000. Nucleic Acids Res 28:45–48. doi:10.1093/nar/28.1.45. 10592178 PMC102476

[10] Tatusov RL, Fedorova ND, Jackson JD, Jacobs AR, Kiryutin B, Koonin EV, Krylov DM, Mazumder R, Mekhedov SL, Nikolskaya AN, Rao BS, Smirnov S, Sverdlov AV, Vasudevan S, Wolf YI, Yin JJ, Natale DA 2003 The COG database: an updated version includes eukaryotes. BMC Bioinformatics 4:41. doi:10.1186/1471-2105-4-41. 12969510 PMC222959

[11] Kanehisa M, Goto S, Kawashima S, Okuno Y, Hattori M 2004 The KEGG resource for deciphering the genome. Nucleic Acids Res 32:D277–D280. doi:10.1093/nar/gkh063. 14681412 PMC308797

[12] UniProt Consortium. 2010 The universal protein resource (UniProt) in 2010. Nucleic Acids Res 38:D142–D148. doi:10.1093/nar/gkp846. 19843607 PMC2808944

[13] Altschul SF, Gish W, Miller W, Myers EW, Lipman DJ 1990 Basic local alignment search tool. J Mol Biol 215:403–410. doi:10.1016/S0022-2836(05)80360-2. 2231712

